# 'We’re all in the same boat … some of us just have more holes in their boat': a qualitative interview study of primary care staff views of Deep End Cymru

**DOI:** 10.3399/BJGPO.2025.0019

**Published:** 2025-12-19

**Authors:** Louise Thompson, Kathrin Thomas, Haroon Ahmed, Fiona Wood

**Affiliations:** 1 Division of Population Medicine, Cardiff University, Cardiff, Wales; 2 Deep End Cymru, Wales; 3 PRIME Centre Wales, Cardiff University, Cardiff, Wales

**Keywords:** qualitative research, inequalities, doctors' health, general practice, primary health care

## Abstract

**Background:**

Socioeconomic deprivation is associated with lower life expectancy and more complex health needs. General practices may mitigate some of these health impacts by providing holistic care to their patients. The Deep End network was established in 2009 in Scotland to support practices working in the most socioeconomically deprived communities, and the concept has since spread, with Deep End Wales (Cymru) launching in 2022.

**Aim:**

To explore experiences of staff working within Deep End practices in Wales and understand their motivations for choosing to work in challenging practices along with their needs from a Deep End network.

**Design & setting:**

Qualitative study with staff from Deep End eligible practices in Wales.

**Method:**

Seventeen semi-structured interviews were undertaken. Data were analysed using thematic analysis and interpreted with reference to self-determination theory.

**Results:**

The following four main themes were identified: (1) Treading water (experiences of providing care in Deep End practices); (2) Diving into the Deep End (motivations for working in a Deep End practice); (3) Providing a life jacket (support from the Deep End Cymru community); and (4) Swimming to shore (the search for work-based effectiveness).

**Conclusion:**

Deep End staff reported high workload, with limited resources and time to manage complex health needs. Most participants found working in Deep End practices rewarding. However, there were concerns about staff burnout, recruitment, and retention of staff. Deep End Cymru is providing hope, validation, and a place to share ideas. Barriers to success were funding and time. Participants want Deep End Cymru to advocate for them, support recruitment, improve services for patients, and support research.

## How this fits in

The Deep End network has been set up to support and advocate for general practices that serve communities facing additional challenges that come with socioeconomic deprivation. Informed by self-determination theory, we explored the experiences of primary care staff working in Deep End communities, what motivates them to continue working, and how the Deep End network could support them. Despite their daily challenges and frustrations, we found that intrinsic motivation for staff was fuelled by a desire to help their community and their experiences of making a difference to their patients. The network is a source of support for GPs to share and validate their experiences as well as providing hope and advocacy for their work.

## Introduction

Socioeconomic deprivation is linked to shorter life expectancy, and higher rates of complex health needs.^1,2^ Social determinants of health that together negatively affect health and wellbeing throughout the life course include a range of non-medical factors at various levels, which influence health conditions such as social and community networks, access to quality education, employment and housing, food security, and working conditions.^3^ The Deep End approach was inspired by Julian Tudor Hart, who coined the term *'Inverse Care Law — that the availability of good medical care varies inversely with population need'*, back in 1971.^4^ ‘Deep End’ refers to the additional needs for populations living in the most deprived areas and the greater workload and complexity for general practices that support these communities.

The first Deep End network was established in Scotland in 2009.^5^ Since then, numerous Deep End networks have been set up.^6–9^ The purpose of the networks is to enable staff to advocate for themselves and their patients, to reduce risk of practitioner burnout, and to develop grassroot interventions. The Royal College of General Practitioners in Wales hosted the first Deep End network in Wales (Deep End Cymru) in 2022, funded by the Welsh Government. The initial 18-month phase was to establish whether Deep End is feasible, acceptable, and supported in Wales. In the first year, 85% of the 100 most deprived practices responded positively to an invitation to be involved.^7^ The key issues identified by members included funding, recruitment and retention, mental health, patient literacy and advocacy, complaints and low morale, older people and comorbidities, and education and training.

Self-determination theory can help to explain human motivations in the workplace postulating that staff have three basic psychological needs for: competence, autonomy, and relatedness.^10^ If these are met, then staff perform better and have improved wellbeing. We aimed to explore experiences of frontline staff who are working in the general practices serving the most deprived communities in Wales through a lens of self-determination theory, with particular emphasis on the unique challenges they face in the Welsh context and their motivations for both working in challenging environments and for participating in the network.

## Method

### Study context

**Figure 1.**
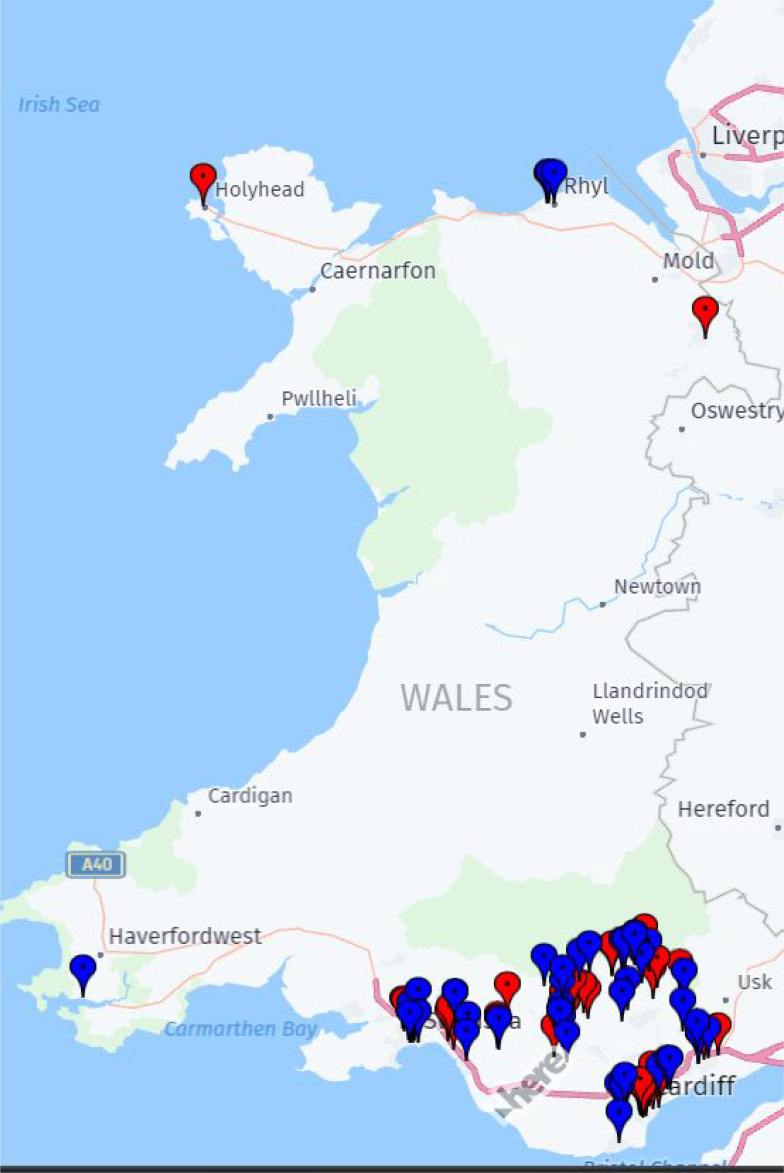
Map of Deep End Cymru practices. Red (lighter) pins = top 50 for percentage of patients in most deprived 20%. Blue (darker) pins = top 51–100 for percentage of patients in most deprived 20%.

The setting for this project was general practices serving the most deprived communities in Wales. Deep End practices were identified using a similar approach to Deep End Scotland: the 389 practices in Wales were ranked by the percentage of their registered patients living in the 20% most deprived Lower Super Output Areas (LSOAs; at January 2022). The highest ranking 100 practices were invited to join Deep End Wales from September 2022. Practices had at least 34% of patients living in the most deprived LSOAs. These practices were concentrated in Swansea, Cardiff, Newport, and the post-industrial South Wales Valleys towns. See Figure 1.

### Design

We conducted an explorative qualitative study using semi-structured interviews, informed by previous research undertaken in the North East and North Cumbria Deep End.^9^ Interviews were conducted between September 2023 and May 2024.

### Participants and recruitment

Participants were provided with a participant information sheet and consent form prior to the interview. Signed consent forms were returned by email. Transcripts were anonymised and kept securely on university servers. Individuals were eligible to participate if they worked in one of the 100 Deep End eligible practices. Participants did not need to have engaged in Deep End Cymru activities to be part of the research. We used a purposeful recruitment strategy to allow for a range of professional backgrounds, experiences, and localities within Wales. We used snowball sampling to boost recruitment.

Eligible practices were invited to participate via an email from the Deep End Project Manager. The lead researcher (LT) also attended Deep End Cymru events to recruit further.

Our initial sample size was guided by the concept of ‘information power’.^11^ We considered a sample size of 15–20 participants to be adequate, informed by previous experience of similar research.^6,9^ Data saturation was reached when no new themes were identified from the data.

### Data collection and analysis

Semi-structured interviews were audio-recorded on Microsoft Teams and audio files were stored securely, transcribed verbatim, and transcripts anonymised. Demographic data were collected to describe the sample: locality, practice size, length of experience in primary care, age group, and sex. Following discussion in early interviews, we added a question regarding research in Deep End practices.

We used a reflexive thematic analysis approach^12^ supported by Nvivo (version 14) data analysis software. An iterative approach was used with the first author generating initial codes while being mindful to search for data that departed from dominant accounts. A second researcher (FW) also coded five transcripts after which discussions about the coding and theme development were held in order to explore alternative interpretations. Codes were sorted into potential themes using mind maps. The team also met to review themes, ensuring themes were coherent and data supporting them were adequate.

Interviews were undertaken by the first author (LT) who works as a GP and has worked in Deep End practices; however, they were not directly involved with Deep End Cymru. The other researchers have backgrounds including public health, general practice, and medical sociology. One of the authors (KT) is directly involved in Deep End Cymru network.

## Results

Seventeen participants were interviewed. The interviews lasted between 34 and 73 minutes (mean: 48 minutes). Of the 17 participants, 12 were GP partners or had worked as salaried GPs, four were practice managers, and one was an allied health professional. Most participants were based in South Wales and had worked in primary care for more than 10 years. Participant characteristics are detailed in Table 1.

**Table 1.** Characteristics of participants

Participant characteristic	*n* = **17**
**Sex**	
Female	14
Male	3
**Age, years**	
<35	2
35–55	10
>55	5
**Health board within Wales**	
Aneurin Bevan (South East Wales)	6
Betsi Cadwaladr (North Wales)	1
Cardiff and Vale	5
Cwm Taf (post-industrial towns)	2
Swansea Bay	3
**Professional background**	
GP partner	10
Practice manager	4
Salaried or locum GP	2
Allied health professional	1
**Length of experience**	
<10 years	5
10–20 years	6
>20 years	6

The following four main themes were identified reflecting their challenges and work-based motivations: (1) Treading water: experiences of providing care in Deep End practices; (2) Diving into the Deep End: motivations for working in a Deep End practice; (3) Providing a life jacket: support from the Deep End Cymru community; and (4) Swimming to shore: the search for work-based effectiveness.

### Treading water: experiences of providing care in Deep End practices

Participants discussed the complexity of the population they served with high rates of mental illness, drug and alcohol abuse, domestic violence, and complex chronic illness. Patients often experienced poor housing, unemployment, and low income, all of which contributed to their health issues:

*'You’ve kind of got the baggage of a life lived in deprivation and all the impacts that has on your quality of life, and your physical health and your mental health, and multimorbidity*.' (Interview 5)

Most clinicians felt the complexity of the consultations was too much to manage within 10 minutes:

*'There is absolutely no way that you can deliver adequate care in a 10-minute appointment for somebody. You have a patient who, you can see there are issues that need to … that you need to look at, but you’re almost frightened to open that up.'* (Interview 10)

A few felt that they had developed a particular expertise in managing complex patients. However, most felt there was a general lack of resources, and those who needed the help had the least access:

*'We’re all in the same boat … some of us just have more holes in their boat.'* (Interview 4)

Many Deep End practices reported proactive approaches to support patients. Many used mental health workers and community connectors. Some practices reported additional services for patients experiencing homelessness and asylum seekers, some had reached out to the community to improve vaccination rates, some changed their websites so it could be accessed in different languages, and many had upskilled in areas such as contraception and substance misuse.

There were mixed feelings towards working in clusters (groups of practices that work together locally to develop services).^13^ Those who found it helpful tended to work with practices that had a similar population. There was a general feeling that services worked well when set up locally, but services were often regional rather than local:

*'... they can’t afford the bus to get over there, they can’t afford a taxi so no one goes … people can’t access the health care they need.'* (Interview 6)

Participants reported a mismatch between supply of appointments and demand, leading to frustration and sometimes abuse from patients. The demand was felt by both clinicians and administrative staff. Many participants felt helpless and were seeing high rates of burnout. This made it harder to recruit and retain staff, leading to even further staff shortages:

*'There are high levels of burnout in the GP population in general, but I think it’s particularly intense in Deep End practices, where you know, GPs are struggling, and they’re massively under-resourced*.' (Interview 16)

There were ongoing concerns that often there were only one or two GPs holding practices together:

*'I do worry even more now, about these sort of Valleys practices and these ones are all struggling, um, how the future is going to be for them.'* (Interview 15)

Some practices reported being actively involved in research, a few found it helpful financially, but often the motivation was contributing to research that benefitted their patients. They viewed their population as enthusiastic and willing to take part in research. Other practices felt that it was not a priority stating they had too much on their plate.

### Diving into the deep end: motivations for working in a Deep End practice

All participants gave altruistic reasons for working in Deep End practices. They described their desire to help and reflected on the moral imperatives that everyone was entitled to equal health care:

*'I’ve always sort of been brought up to have that sense of like everyone’s equal … I just think like morally, everyone should have that access to health care that they need.'* (Interview 4)

Almost all participants felt it was rewarding to work in a Deep End practice, and they would not necessarily get the same job satisfaction from working in more affluent areas:

*'A lot of the time it’s, you feel like you’re actually making a difference ... which I guess is what we all want to do in this world isn’t it.*' (Interview 1)

Although in some practices there had been challenges dealing with frustration from patients and sometimes abuse of staff, many participants enjoyed working with their population usually finding patients very grateful for the help they received. Many participants also enjoyed the diversity of the populations they served, and the range of problems they encountered, finding they were usually seeing patients that needed help.

Many participants enjoyed working at their practice owing to the practice culture and a supportive environment. A few mentioned they trained at the practice and decided to stay on once qualified; this was also seen to aid recruitment in these practices:

*'I think there’s a reason I wanted to stay is because it’s got a particular culture of support and it’s a training practice, we’ve got students so the whole thing is positive even in difficult circumstances.'* (Interview 6)

### Providing a life jacket: support from the Deep End Cymru community

Most participants were from practices that had engaged in some way with Deep End Cymru. Three participants mentioned limited or no involvement.

Many participants involved in Deep End Cymru felt it was a validating experience:

*'We all know that this has been going on for a long time. But I think for the Deep End project, it’s you know, it’s got a name and … and we can all sort of … I don’t know, you’re stronger together, with these things.'* (Interview 4)

There was a sense that being able to come together with people who understood the challenges was a source of support and one of the main positive impacts:

*'Some of the ideas coming through are easily transferrable and really good, and what other people are doing. For me, it’s more about the community I think, and that sharing of things that have worked really well, or you know, the outcomes for the individuals as well.'* (Interview 2)

Many voiced how important they felt Deep End Cymru was, and that by having a network, even in its early days, was a source of hope and support. They explained that it made them feel less isolated and gave hope that things may improve. There was also a sense that they were coming together with like-minded professionals who all wanted the best for their patients:

*'The sort of want for, for helping the people, their patients and getting the best for their patients I think, was the, one of the biggest take-homes from it.'* (Interview 8)

There were barriers to engagement. Participants felt they were ‘firefighting’ every day, so some did not feel they could spare staff to attend meetings. Participants voiced they had no *‘*headspace*’* to think about improvements. Another barrier was money. Deep End Cymru provided payment to allow a member from each practice to attend meetings. Many said this was not enough to provide cover and that these barriers would affect the practices that were struggling the most. Those that had not engaged with Deep End Cymru may be the ones that needed the most help:

*'It’s a ‘Catch 22’ isn’t it, a network always means that you are engaging, that always means time and we are asking time of practices which are the ones struggling the most.'* (Interview 13)

### Swimming to shore: the search for work-based effectiveness

All participants discussed the need to change how primary care is funded with many feeling that the current Carr-Hill formula^14^ did not take into consideration levels of deprivation adequately. This was felt to be inequitable when considering the increased workload and how often practices struggled to meet their targets:

*'The Carr-Hill formula does not work for us. It’s making us look like we do less work, but we do more.'* (Interview 11)

Participants felt one important way in which the network could support practices was in advocacy. It was felt there was a general lack of awareness (including from Welsh Government, other practices, academics, and the public) of the difficulties they faced. Some participants also felt it would be helpful to get the public involved when advocating for Deep End practices:

*'I think one of the most positive things it can do is campaign to get better funding because unfortunately money makes the world go round.'* (Interview 1)

With increased funding, participants felt they would be able to offer better services including more and longer appointments. Many spoke about administrative support to allow practices to be more proactive at contacting patients about chronic health reviews and vaccinations. Others discussed employing other staff, such as mental health workers or link workers, to allow a more holistic approach to patient problems. All practices felt that they could spend time improving health literacy. Practices with a high proportion of patients who do not speak English as a first language wanted extra funding to offer patients longer appointments and resources in different language.

*'If you’ve got a link worker, and say right, let’s look at the housing, let’s try and support you through that bit, and it might not work, but at least when we get through that, we can do the kind of broader piece.'* (Interview 5)

Many participants wished for more protected time and headspace to implement solutions rather than firefighting. In order to achieve this, they wanted help with recruitment and retention of staff. They feared that some practices are unable to continue and are resigning their contracts. Some felt this could be done by increasing student placements and GP trainees to Deep End practices. Many were enthusiastic about the prospect of research, but would need protected time for training of staff, and time and funding to do the research itself.

Most participants were enthusiastic about Deep End Cymru and what it may achieve. However, a few were more cynical. There was a feeling that keeping practices engaged may be difficult unless they saw the benefits of the network and, as part of this, clearer objectives were needed. Others suggested short-term and longer-term outcomes. One suggestion was a measure of the proportion of practices engaged in Deep End Cymru. Many participants suggested staff-related outcomes such as feedback on whether workload had improved, measuring burnout among Deep End staff, and the numbers of practices that resigned their contracts.

*'One big thing is staff, so kind of staff retention and recruitment, so monitoring staff levels in these practices, perhaps you could look at you know, the number of patients per partner, and if that’s rapidly rising, that suggests that practices are struggling*.' (Interview 16)

Patient-related success outcomes suggested were repeat appointment levels, which may indicate that patients’ needs are being met within one consultation, and rates of behaviours such as smoking, drinking alcohol, or immunisation uptake. Longer-term outcomes discussed were life expectancy of patients in Deep End practices, and whether chronic illness rates had improved. Other suggestions were looking at whether practices were financially better off.

## Discussion

### Summary

This study explored participants’ experiences of working in Deep End Practices in Wales and of the Deep End Cymru network through a lens of social determination theory. Participants emphasised the complexity of the populations they supported, and their frustrations that they did not have adequate resources or time to provide high-quality holistic care. Demand and workload were felt to be high. There were concerns raised about staff burnout, and recruiting and retaining staff. Despite these challenges, many participants enjoyed working in Deep End practices, often finding the work rewarding and having good working relationships with their practice teams. The importance of continuity of work from trainee to qualified GP within the same practice was stressed. Most participants engaged with Deep End Cymru and felt the network was offering hope, validation, and a place to share ideas. Thus, participants' intrinsic motivations for continuing to work in challenging circumstances were mitigated by their desire to contribute meaningfully. Furthermore, the Deep End network appears to be providing support through strengthening feelings of belonging, buffering the impact of stress, and fostering fulfilment.

Although participants were generally positive about Deep End Cymru, there were concerns about barriers to engagement; the main ones being funding, and time to attend meetings and make changes. Participants hoped that Deep End Cymru could advocate for the practices and for changes to the funding of primary care, which would better reflect the needs and increased workload. They also hoped that the network could help with recruitment, improve services for patients, increase awareness to the problems they face, and support research.

### Strengths and limitations

By using semi-structured interviews, we ensured participants were asked similar topics, but with scope for deeper exploration and understanding of participants’ views about issues of importance to them. Interviews were conducted by a single researcher, who, although working as a GP in a Deep End practice, was not previously involved with Deep End Cymru. This allowed for participants to be more open about the network.

The researchers aimed for a breadth of participant characteristics. Although participants who agreed to be interviewed were a self-selected group who were interested in Deep End Cymru. We were only able to recruit one allied health professional. This may limit whether findings are applicable to all primary care staff in Deep End practices.

### Comparison with existing literature

Other studies have previously reported that GPs choose to work in Deep End environments as they are both stimulating and rewarding.^8^ Our data confirm this, identifying that GPs find intrinsic satisfaction from their work with their communities. However, we have also identified that the construct of relatedness within self-determination theory, that is feelings of belonging to a social group such as the Deep End network, is of importance. Many of our participants were motivated to work in Deep End practices owing to finding the work rewarding and wanting to help tackle health inequalities. Babbel *et al* ^15^ interviewed GPs working from socioeconomically deprived areas in Scotland and found two main belief systems among participants: some stressed the need to address wider social determinants of health to help tackle health inequalities; while others were more likely to put the onus of behaviour change onto individual patients. Interestingly, those GPs who showed more empathy for their patients’ social circumstances were more likely to be involved in the local Deep End network.

Similar to research conducted in other Deep End networks,^9^ our participants felt that the resources available were not meeting the needs of their patients and that the 10-minute consultation is inadequate to manage complexity. Almost all participants voiced their concerns about the way primary care is funded and that Deep End practices were disadvantaged by the funding system.^16,17^ A key finding from the North East and North Cumbria Deep End was the idea that general practice is expected to address health inequalities, without the resources to do so, especially in the areas that need it most.^9^ This was a message that many of the participants from our study also reiterated.

The EQUALISE study^18^ concluded that evidence is poor on how GPs can impact health inequalities. Together with the FAIRSTEPS project, a practical toolkit has been developed.^19^ A key finding was that increased funding in primary care, especially to areas of high socioeconomic need, can increase staff and capacity, leading to an increase in primary prevention in high-risk patients and improving health outcomes. Another recommendation is that interventions should be tailored to specific patient populations. This was a common finding throughout our interviews, with interventions set up regionally either not being accessible through structural barriers such as transport, more difficult to access owing to social aspects of the patients’ lives, or even language barriers that weren’t taken into consideration, limiting the effectiveness for certain groups of patients.

Many participants were positive about the impact that Deep End Cymru had made so far, feeling it provided a place to share ideas, and was offering validation and hope for change in the future. This is in keeping with research conducted in other Deep End networks.^16^ Some felt that Deep End Cymru needed to develop clearer aims and objectives to engage more eligible Deep End practices. The stakeholders’ analysis conducted by Deep End Cymru ^20^ agreed with many of these findings, but some participants felt the progress to date was slow, which is likely owing to barriers of time and funding.

### Implications for research and practice

We acknowledge that the inclusion of views from the wider primary care team would be helpful, and this will require engagement and funding.

We suggest that there are three fruitful areas for further research: evaluation of delivery of the Deep End Programme itself; building capacity and infrastructure across Deep End practices for research delivery; and generating and conducting new research of relevance to Deep End populations.

Participants felt that the success of the network could thus be measured by GP engagement, reduction in burnout levels, increased recruitment and retention, and patient outcomes such as chronic disease reviews and vaccination rates. In the longer term, the network’s legacy could be in reducing chronic illness and increasing the life-expectancy of their populations. This would enable Deep End Cymru to demonstrate impact to secure further funding.

The main barriers are funding and protected time to participate. Some eligible GP practices may not be aware of what the network can offer. The inequity of funding and the use of the Carr-Hill formula was a common theme. Suggestions from participants included advocating for their practices and transparency of data that demonstrates this. There was also felt to be a need for improved and accessible patient services.

The evaluation in 2024 found that 43% of Welsh Deep End practices had training status, compared with 58% in the two most affluent groups of GP practices.^7^ Future research may explore how best to prepare health professionals to work in Deep End practices through existing training schemes by including deprivation medicine or setting up specific deprivation medicine training schemes that have been successful elsewhere.^21,22^

Our qualitative analysis has underscored the challenges that GPs at the Deep End face, their motivations, and their hopes for the Deep End Cymru network. It has also identified some reasons for not engaging with the network. There seems to be an appetite for a network to enable these Deep End practices to survive and flourish for the benefit of the communities they serve.

## Data

The dataset relied on in this article is available from the corresponding author on reasonable request.
